# Evolutionary dynamics of emblematic *Araucaria* species (Araucariaceae) in New Caledonia: nuclear and chloroplast markers suggest recent diversification, introgression, and a tight link between genetics and geography within species

**DOI:** 10.1186/s12862-014-0171-6

**Published:** 2014-09-05

**Authors:** Myriam Gaudeul, Martin F Gardner, Philip Thomas, Richard A Ennos, Pete M Hollingsworth

**Affiliations:** UMR CNRS-MNHN-UPMC-EPHE 7205 ‘Institut de Systématique, Evolution, Biodiversité’, Muséum National d’Histoire Naturelle, 16 rue Buffon, CP 39, F-75005 Paris, France; Royal Botanic Garden Edinburgh, Edinburgh, EH3 5LR United Kingdom; Institute of Evolutionary Biology, University of Edinburgh, Ashworth Laboratories, Edinburgh, EH3 9JT United Kingdom

**Keywords:** Admixture, Closely related species, Conifers, Diversification, Hotspot, Hybridization, Introgression, Phylogeography, Population genetics, Systematics

## Abstract

**Background:**

New Caledonia harbours a highly diverse and endemic flora, and 13 (out of the 19 worldwide) species of *Araucaria* are endemic to this territory. Their phylogenetic relationships remain largely unresolved. Using nuclear microsatellites and chloroplast DNA sequencing, we focused on five closely related *Araucaria* species to investigate among-species relationships and the distribution of within-species genetic diversity across New Caledonia.

**Results:**

The species could be clearly distinguished here, except A. *montana* and *A. laubenfelsii* that were not differentiated and, at most, form a genetic cline. Given their apparent morphological and ecological similarity, we suggested that these two species may be considered as a single evolutionary unit. We observed cases of nuclear admixture and incongruence between nuclear and chloroplast data, probably explained by introgression and shared ancestral polymorphism. Ancient hybridization was evidenced between *A. biramulata* and *A. laubenfelsii* in Mt Do, and is strongly suspected between *A. biramulata* and *A. rulei* in Mt Tonta. In both cases, extensive asymmetrical backcrossing eliminated the influence of one parent in the nuclear DNA composition. Shared ancestral polymorphism was also observed for cpDNA, suggesting that species diverged recently, have large effective sizes and/or that cpDNA experienced slow rates of molecular evolution. Within-species genetic structure was pronounced, probably because of low gene flow and significant inbreeding, and appeared clearly influenced by geography. This may be due to survival in distinct refugia during Quaternary climatic oscillations.

**Conclusions:**

The study species probably diverged recently and/or are characterized by a slow rate of cpDNA sequence evolution, and introgression is strongly suspected. Within-species genetic structure is tightly linked with geography. We underline the conservation implications of our results, and highlight several perspectives.

**Electronic supplementary material:**

The online version of this article (doi:10.1186/s12862-014-0171-6) contains supplementary material, which is available to authorized users.

## Background

Over the last decades, both theoretical and empirical investigations have underlined the continuum of evolutionary processes acting on populations and species (e.g. [1-5]). Therefore, an increasing number of studies adopt an integrative approach and combine concepts, molecular techniques and statistical tools from both areas to shed light on the evolutionary dynamics of closely related species. In such groups, species may undergo a process of divergence (with population differentiation as the first step) and reproductive isolation and, in different places or at different times, may exchange genes through hybridization and introgression if reproductive isolation is not complete. The speciation process lies at the heart of this interface between population genetics and phylogenetics, with many questions that remain unanswered in most cases e.g. when and why did populations initially diverge? What were their relative geographic distributions (sympatry *vs.* allopatry)? How did reproductive isolation emerge? Such questions are crucial challenges faced by biologists to understand the evolution of species, and to better preserve the mechanisms generating diversity in the face of today’s major environmental threats.

New Caledonia is a biodiversity hotspot [6] with more than 3000 native angiosperm and 43 conifer species in an area of ca. 19000 km2. It is characterized by high endemism (77% for angiosperms and 100% for conifers; [7]). Although it is of continental Gondwanan origin and geographically isolated (1500 km east of Australia), recent studies have shown that the modern New Caledonian biodiversity largely originates from dispersal events and in situ species radiations [8,9]. This is congruent with the long submersion of the island (ca. 65–37 Ma; [10,11]), and radiations were probably favoured by environmental gradients –notably in terms of soil substrate, altitude and climate– which have created a variety of habitats within a small geographic area. Also, in contrast to what was long supposed given the old separation of New Caledonia from Australia (ca. 80 Ma; [11]) and congruent with the submersion of the island, most radiations would be relatively recent (< 15Ma; reviewed in [12,13]). In this context, studies at the interface between population genetics and phylogenetics seem best suited to examine how the biodiversity of New Caledonia emerged. However, to date, most investigations have dealt with taxonomy and large-scale phylogeny (e.g. [14-17]) –reflecting our very incomplete knowledge of the flora and fauna and justified by the strong pressure of habitat destruction due to human-set fires and open-cast mining activities [18,19]. Only a few plant studies have examined relationships among closely related species [20-24] or surveyed the distribution of within-species genetic diversity on the island, with the latter often focused on a low number of populations or small geographic area [25-30]. Yet, surveys of population genetics also provide information on the recent history and could be useful to detect the possible impacts of the Quaternary climatic oscillations experienced by the New Caledonian flora: in the southeast of the island, palynological records suggested that vegetation alternated between rainforest and maquis from 120,000 to 50,000 yr ago, and a compelling Araucaria decline was detected around 45,000 yr ago [31]. However, the location of potential refugia remains highly uncertain (but see [30,32]). Populations that originate from the same refugia are expected to form a genetically more or less homogeneous group compared to populations that colonized from distinct refugia [33]. Nevertheless, given the complex topology of the island, with many physical barriers, genetic drift could have a strong influence on the genetic structure relative to current gene flow, and cause significant differentiation among populations.

Here, we studied species of the conifer genus *Araucaria* Juss. (Araucariaceae) and were both interested in among-species relationships and the distribution of within-species genetic diversity across New Caledonia. The genus *Araucaria* comprises a total of 19 species worldwide, 13 of which are endemic to the archipelago. They usually occur as large populations, in a variety of ecological habitats (most often humid forest or maquis; [34]). Importantly, all species are confined to ultramafic soils –characterized by low fertility (low N, P, K), high concentrations of heavy metals (e.g., Co, Cr, Ni) and low water-holding capacity [35]– except *A. montana* Brongn. & Gris, which occurs on both ultramafic and non-ultramafic soils and *A. columnaris* Hook. and *A. schmidii* de Laub., which only occur on calcareous and acidic soils, respectively. *Araucaria* trees are long-lived, monoecious trees whose breeding system is largely unknown. Pollen dispersal is wind-mediated and probably larger than seed dispersal, which mainly occurs by gravity. However, secondary seed dispersal by animals and strong winds is likely since they possess wings and ripen during the cyclone season [36].

Based on rbcL sequencing, Setoguchi et al. [37] showed that New Caledonian *Araucaria* species form a monophyletic group and, using AFLP markers, Gaudeul et al. [34] delineated three clades within this group: the large-leaved, small-leaved and coastal clades. Nevertheless, the exact relationships among species remain unresolved, especially within the large-leaved group that includes six species: *A. montana*, *A. laubenfelsii* Corbasson, *A. rulei* F.Muell., *A. biramulata* J. Buchholz, *A. muelleri* (Carrière) Brong. & Gris and *A. humboldtensis* J. Buchholz. In addition, the distinction of *A. montana* and *A. laubenfelsii* –described in 1871 and 1968, respectively [38]– was questioned by field observations: they have very similar morphology and ecology and their distribution areas are contiguous, with *A. montana* being found in the north while *A. laubenfelsii* is restricted to some localities in the south. As for many New Caledonian plant species, several *Araucaria* populations/species are threatened by habitat destruction [28,39]. However, the reduction of suitable habitat and population size has probably not left any signature in the genetic structure of today adult populations, because *Araucaria* trees are very long-lived (> 500 years; [40]) and habitat destruction comparatively recent (starting in 1874; [39]). Consequently, most adult trees observed today existed before the beginning of habitat destruction, and there has been little opportunity for changes in genetic structure of remaining populations since then. Previous genetic surveys on New Caledonian *Araucaria* species focused on the highly threatened and geographically restricted *A. nemorosa* and its widespread relative *A. columnaris* [28,41,42]. These studies covered a small geographic area, and were primarily aimed at providing practical information for conservation.

We examined the genetic structure of four species that are largely distributed in New Caledonia: *A. montana* and *A. laubenfelsii* (which may form a single species), *A. rulei*, and *A. scopulorum* (which belongs to the coastal clade whereas the other three belong to the large-leaved clade). Also, some species sometimes grow sympatrically and we suspected introgression, which may account for the difficulty to resolve the species phylogeny. Since *A. biramulata* may be involved in such events based on our field observations (intermediate morphology and unusual ecology of a given population, in Mt Tonta), it was also surveyed here. Ancient hybridization events and introgression may be detected through genetic admixture at nuclear loci (see e.g. [43]), or through incongruent genealogical patterns between uniparental and nuclear DNA (e.g. [3]). The retention of ancestral polymorphism, or ‘incomplete lineage sorting’ , can lead to similar incongruence and disentangling the two phenomena –that are both more likely among closely related than anciently diverged species– is sometimes difficult (see e.g. [5,43-45] and references therein). Retention of ancestral polymorphism is especially likely when the effective sizes of diverging species are large and substitution rates are low [1,46]. Hybridization and introgression require incomplete reproductive isolation, and are obviously more likely when species grow together in a given (or in spatially close) geographic site(s). Our objectives were: i) to assess whether *A. montana* and *A. laubenfelsii* are genetically differentiated, and whether it is justified to treat them as two distinct species based on such data; ii) more broadly, to estimate the extent of among-species genetic variation and give some insight into their relationships; iii) to search for possible signs of introgression between species; iv) at the within-species level, to examine the spatial structure of genetic variability in order to infer the history of populations and major processes influencing their evolution (e.g. location of potential refugia and relative influence of gene flow *vs.* genetic drift).

We surveyed genetic variation on an extensive population sampling, using both nuclear (nDNA) microsatellite loci and chloroplast DNA (cpDNA) sequencing. Whereas the nuclear genome is diploid and biparentally inherited, the chloroplast genome is effectively haploid and uniparentally, paternally inherited in gymnosperms. Therefore, both genomes are dispersed through seeds and pollen but the chloroplast genome has a smaller effective population size, which makes it more sensitive to genetic drift and population/species differentiation [47]. Moreover, cpDNA is characterized by a lower mutation rate than nDNA, especially when comparing cpDNA intergenic regions to nDNA microsatellites that are highly variable markers.

## Methods

### Plant material

Plant material was collected from 1999 to 2008 (see Additional file 1, Figure 1). Within each site, herbarium specimens were collected for taxonomic identification based on morphological characters (following [38]), together with 2 to 48 silica-dried leaf samples (mean ± S.D.: 21.7 ± 8.4). Sampling sites covered the entire range of species and included the vast majority of existing populations for *A. montana*, *A. laubenfelsii*, *A. biramulata* and *A. scopulorum. Araucaria montana* was sampled both on non-ultramafic (in Mt Panie and TonNon) and ultramafic soil substrates (in all other sites). The natural distribution of *A. scopulorum* is characterized by a disjunction between northwestern and eastern populations. In contrast, some (unsampled) *A. rulei* populations occur between the northernmost and more southern localities surveyed.

**Figure 1:**
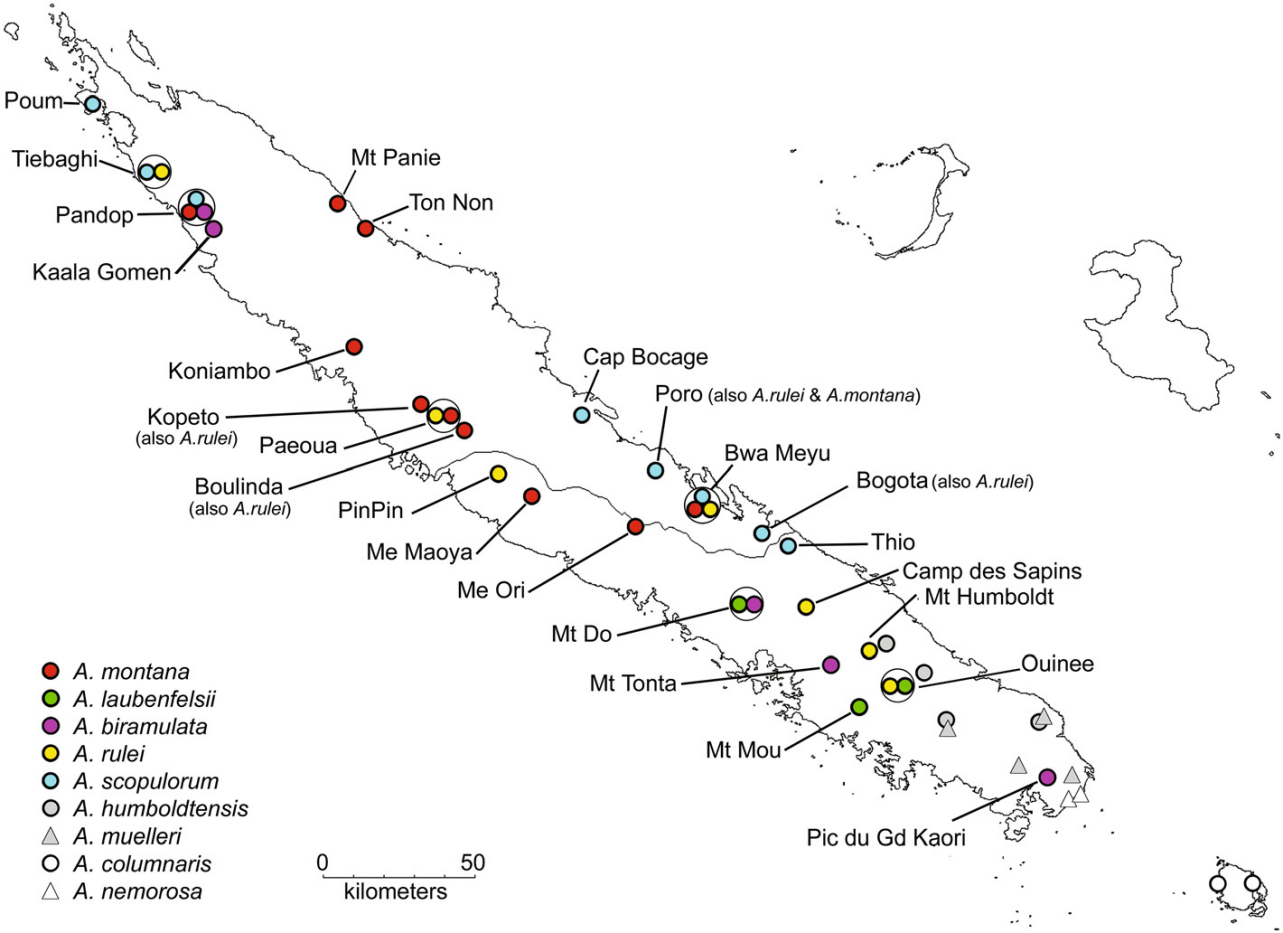
**Map of the study populations.** Dot colours follow the taxonomic identification of populations based on morphology. Sites where several species were studied are circled. Sites where several species co-occur but only one was studied are also identified, and the name of the unstudied species is mentioned.

In two cases, the taxonomic identification was uncertain: i) in Pandop, close inspection of (very partial) herbarium vouchers identified the species as *A. biramulata* in almost all cases (that is, 18 samples), whereas five samples were identified as *A. montana*; ii) in Mt Tonta, trees were identified and considered here as *A. biramulata* but they appeared morphologically somewhat intermediate between *A. biramulata* and *A. rulei*, although the ecological characteristics were more typical of *A. biramulata*.

In total, we studied 711 samples from 32 populations and five species for nDNA (Table 1). For cpDNA, we added a few samples of *A. muelleri* and *A. humboldtensis*, as well as of *A. columnaris* and *A. nemorosa* that belong to the third *Araucaria* clade ([34]; Table 1). In total, the cpDNA dataset included 617 individuals from 45 populations and nine species.

**Table 1:** **Statistics on the genetic diversity and structure of the study populations based on nuclear and chloroplast datasets**

**Species or population**	**Number of samples studied for nDNA**	***H***_**E**_	***AR*** **(based on a minimal size of 3 indiv.)**	***F***_**IS**_	**Mean pairwise** ***F***_**ST**_ ** & species-level** ***F***_**ST**_ **(with/without locus Aru1)**	**Number of samples studied for cpDNA**	**Number of cpDNA haplotypes**	***HR*** **(based on a minimal size of 6 indiv.)**	***MNbPwDiff***	**Mean pairwise** ***N***_**ST**_ **& species-level** ***N***_**ST**_
***A. montana-A. laubenfelsii***	274	0.793 ± 0.126	3.98 ± 0.73	0.116 ± 0.082	0.105/0.109	213	28 (26 specific)	4.04 ± 1.48^ab^	1.152 ± 0.487^a^	0.203
TonNon	24	0.516	2.33	0.190	0.246 ± 0.036	23	1	1.00	0.000	0.318 ± 0.139
Mt Panie	10	0.540	2.66	0.081	0.222 ± 0.044	10	3	2.85	1.133	0.263 ± 0.092
Pandop	2	-	-	-	-	2	1	-	-	-
Koniambo	48	0.842	4.13	0.206*	0.114 ± 0.058	32	6	4.11	1.462	0.283 ± 0.061
Kopeto	6	0.840	4.40	0.206	0.094 ± 0.095	6	3	3.00	0.933	0.175 ± 0.156
Paeoua	26	0.860	4.40	0.194*	0.085 ± 0.064	16	6	5.04	1.775	0.092 ± 0.095
Boulinda	10	0.827	4.02	−0.023	0.129 ± 0.080	10	3	2.85	0.933	0.170 ± 0.155
Me Maoya	24	0.902	4.73	0.132*	0.077 ± 0.069	16	8	6.24	1.817	0.102 ± 0.101
Me Ori	24	0.859	4.41	0.015	0.082 ± 0.072	15	7	5.47	1.524	0.130 ± 0.122
Bwa Meyu	26	0.826	4.13	0.079	0.082 ± 0.063	20	4	3.55	0.884	0.131 ± 0.093
Mt Do	25	0.840	4.28	0.082	0.079 ± 0.069	25	6	3.94	0.947	0.162 ± 0.121
Mt Mou	25	0.816	4.08	0.030	0.091 ± 0.071	22	8	5.50	1.234	0.306 ± 0.164
Ouinee	24	0.849	4.25	0.194*	0.075 ± 0.068	16	7	4.95	1.183	0.240 ± 0.162
***A. biramulata***	86	0.750 ± 0.092	3.58 ± 0.56	0.107 ± 0.059	0.151/0.156	87	8 (4 specific)	2.79 ± 0.72^a^	1.032 ± 0.981^ab^	0.433
Pandop	21	0.812	3.99	0.136*	0.126 ± 0.094	21	2	1.76	0.181	0.211 ± 0.311
Kaala Gomen	24	0.828	4.04	0.058	0.130 ± 0.099	23	4	3.03	0.704	0.176 ± 0.280
Mt Do	19	0.735	3.47	0.176	0.168 ± 0.033	19	5	3.41	0.795	0.170 ± 0.258
Mt Tonta	22	0.626	2.83	0.059	0.211 ± 0.008	22	3	2.95	2.446	0.512 ± 0.052
Pic du Grand Kaori	0	-	-	-	-	2	1	-	-	-
***A. rulei***	155	0.805 ± 0.035	4.00 ± 0.20	0.074 ± 0.047	0.105/0.119	107	13 (10 specific)	3.07 ± 1.22^a^	1.143 ± 0.619^ab^	0.108
Tiebaghi	24	0.832	4.12	0.015	0.100 ± 0.017	16	1	1.00	0.000	0.220 ± 0.166
Paeoua	20	0.825	4.11	0.084	0.132 ± 0.026	13	3	2.72	1.359	0.090 ± 0.124
Pin Pin	20	0.823	4.12	0.032	0.117 ± 0.017	15	4	3.59	1.657	0.056 ± 0.072
Bwa Meyu	24	0.784	3.88	0.131*	0.093 ± 0.026	16	6	4.67	1.592	0.197 ± 0.181
CDS	23	0.847	4.25	0.047	0.085 ± 0.018	15	5	4.07	1.200	0.056 ± 0.078
Mt Humboldt	20	0.765	3.78	0.075	0.104 ± 0.034	16	3	2.23	0.608	0.096 ± 0.121
Ouinee	24	0.759	3.71	0.137	0.109 ± 0.035	16	4	3.23	1.583	0.052 ± 0.081
***A. scopulorum***	198	0.774 ± 0.023	3.67 ± 0.18	0.191 ± 0.060	-/0.119	187	23 (21 specific)	4.88 ± 0.58^b^	1.555 ± 0.346^b^	0.293
Poum	24	0.757	3.40	0.277*	0.157 ± 0.050	22	7	4.63	1.307	0.317 ± 0.203
Tiebaghi	24	0.762	3.56	0.207	0.127 ± 0.021	21	6	5.06	1.914	0.298 ± 0.184
Pandop	23	0.824	4.01	0.129	0.106 ± 0.026	22	7	4.37	1.619	0.295 ± 0.205
Cap Bocage	33	0.780	3.75	0.160*	0.105 ± 0.044	31	6	4.49	1.170	0.231 ± 0.205
Poro	24	0.773	3.59	0.197	0.098 ± 0.058	23	7	5.51	1.447	0.203 ± 0.210
Bwa Meyu	24	0.766	3.76	0.146	0.098 ± 0.050	24	6	4.16	1.112	0.281 ± 0.219
Bogota	24	0.749	3.61	0.134	0.137 ± 0.042	23	6	5.00	1.937	0.206 ± 0.120
Thio	22	0.777	3.68	0.279*	0.086 ± 0.038	21	8	5.85	1.933	0.185 ± 0.177
***A. humboldtensis***	0	-	-	-	-	8	2	-	-	-
***A. muelleri***	0	-	-	-	-	7	3	-	-	-
***A. columnaris***	0	-	-	-	-	4	2	-	-	-
***A. nemorosa***	0	-	-	-	-	4	2	-	-	-
**TOTAL**	713	-	145 alleles	-	Overall: 0.143	617	71	-	-	Overall: 0.646

For each species, populations are ordered from North to South below the species-level estimates. *H*_E_: expected heterozygosity; *AR*: allelic richness; *HR*: haplotypic richness; *MNbPwDiff*: mean number of pairwise differences between pairs of individuals. *indicates *F*_IS_ indices that were significantly > 0. All nDNA estimates include locus Aru1, except for *A. scopulorum* populations. CpDNA haplotypes are characterized as ‘specific’ to a species when they only occur in this species. Different letters as superscripts after species means indicate significantly different values.

### Nuclear microsatellites

DNA was extracted using the DNeasy 96 Plant Kit (QIAGEN, Courtaboeuf, France). Seven nuclear microsatellite markers were tentatively amplified [28,48] but two of them (As110 and As152) could not be amplified or gave ambiguous profiles, and were discarded. Also, locus Aru1 failed to amplify in *A. scopulorum*. With the exception of Aru1, which was designed for *A. rulei*, all microsatellites were isolated and developed for *A. subulata*, a species belonging to the same cluster as *A. scopulorum* according to a previous AFLP phylogeny [34]. PCRs were performed in 16 μl containing 1 μl DNA, 1X Taq Buffer, 2.5 mM MgCl_2_, 0.2 mM of each dNTP, 0.2 μM of each primer (one primer per pair was fluorescently labelled), and 0.5 U Taq polymerase (for all loci except Aru1: Taq CORE kit, MP Biomedicals, Illkirch, France; for Aru1: Ampli*Taq* Gold DNA polymerase, Applied Biosystems, Courtaboeuf, France). The reaction profile was: 40 cycles of 30 s denaturation at 95°C, 30 s annealing and 1 min elongation at 72°C, followed by a final elongation step of 10 min at 72°C. Annealing temperature was 50°C for As167 and As190, and 52°C for As25 and As93. For Aru1, annealing was initiated at 62°C, reduced by 1°C for the next 10 cycles, and maintained at 52°C for the subsequent 29 cycles. PCR products were then mixed in a 2/3/6/6/5 ratio for As167/As190/As25/As93/Aru1, respectively and loaded on an automated sequencer (Genoscreen, Lille, France). Microsatellite profiles were manually genotyped using GeneScan 3.7 and Genotyper 3.7. For all five loci, reproducibility was checked by performing the amplification and genotyping steps twice on 38 samples. The error rate was calculated as the proportion of mismatches between duplicated, single-locus genotypes.

### cpDNA sequencing

Three regions were sequenced: a subregion of the intergenic spacer *psb*A-*trn*H, a subregion of the intergenic spacer *trn*S-*trn*fM and the intergenic spacer *atp*H-*atp*I (Table 2). PCRs were carried in 25 μl containing 1 μl DNA template, 1X Taq Buffer, 2.5 mM MgCl_2_, 0.2 mM of each dNTP, 0.2 μM of each primer (except for *atp*H-*atp*I: 0.4 μM), and 0.5 U Taq polymerase (Taq CORE kit). The reaction profile was: 40 cycles of 30 s denaturation at 95°C, 30 s annealing at 50°C (except for *atp*H-*atp*I: 45°C) and elongation at 72°C, followed by a final elongation step of 10 min at 72°C. Elongation time was 30 s for *psb*A-*trn*H, 2 min 30 s for *trn*S-*trn*fM and 2 min for *atp*H-*atp*I. PCR products were then sequenced in both directions at the Centre National de Séquençage (Evry, France). The resulting sequences were manually inspected in Sequencher® 4.9 (Gene Codes Corporation, Ann Arbor, MI, USA), and aligned in MEGA 4.0.1 [49]. Since cpDNA does not experience recombination, we concatenated the sequences of the three genomic regions, so that each sample was characterized by its multi-region haplotype. In order to include both substitutions and microsatellites (mononucleotide repeats) in our analyses, and because computer programs sometimes exclude gaps in calculations, we coded microsatellites as A when a repeat was present and T when it was absent (i.e. in place of gaps).

**Table 2:** **Primers used for cpDNA PCR and sequencing**

**Genomic region**	**Primer name**	**Primer sequence**	**Reference**
partial *psb*A-*trn*H	HAm_F	CCGATGGATTGTTAGTGTGT	-
partial *psb*A-*trn*H	HAm_R	TTCTATGATTTAGAAGAGTCC	-
partial *trn*S-*trn*fM	trnS	GAGAGAGAGGGATTCGAACC	[50]
partial *trn*S-*trn*fM	AP3R	CCCTGGCAAAGAGAAATTTTACC	-
partial *trn*S-*trn*fM	AP1F*	TCCCTCTTCTCTCCCACTCAAAT	-
*apt*H-*atp*I	atpH	CCAGCAGCAATAACGGAAGC	[51]
*apt*H-*atp*I	atpI	ATAGGTGAATCCATGGAGGG	[51]

*only used for sequencing.

### Statistical analyses

Nuclear and chloroplast datasets were analyzed separately. For each dataset, some analyses were performed on the total sampling i.e. simultaneously considering all species, while others were computed within each species separately. For nDNA, among-species comparisons including *A. scopulorum* were run on a dataset excluding locus Aru1 so that the data were comparable.

Based on the results obtained on the multispecies dataset, *A. montana* and *A. laubenfelsii* were considered as a single species in the within-species analyses. The *A. montana* samples from Pandop and *A. biramulata* samples from Pic du Grand Kaori were removed from analyses at the population level because sample sizes were too low (see Results for *A. montana* in Pandop). Last, we did not consider the geographic structure of genetic diversity in *A. biramulata*, because our sampling was too limited and two populations seemed to undergo genetic exchange with other species (see below), precluding reliable within-species inferences.

#### Nuclear microsatellites

##### Multispecies analyses to examine species divergence and relationships

We used Structure version 2.3.3 [52,53] to cluster individual genotypes into *K* genetically distinct clusters. Structure was run ten times for each *K*-value from one to eight to check the consistency of the results across runs. Each run comprised a burn-in of 200000 iterations followed by 10^6^ iterations. We adopted the admixture and correlated allele frequencies models. We used StructureHarvester v0.6.93 [54] to plot the relationship between *K* and (i) the probability of the data L(*K*) and (ii) the ad hoc statistic Δ*K* recommended by Evanno et al. [55]. We identified the most relevant number of clusters as the one that maximized L(*K*) and/or Δ*K*. A similar analysis was run with a reduced dataset, only including *A. montana* and *A. laubenfelsii* populations.

Based on the matrix of *F*_ST_ indices between all pairs of populations (see below), the relationships between populations were visualized using the network-building distance-based algorithm Neighbor-Net [56,57] implemented in Splistree4 [58].

The overall population differentiation was quantified by the estimator *θ* of *F*_ST_ [59], calculated in FSTAT [60], and hierarchical analyses of molecular variance (AMOVAs) were conducted to partition the total genetic variance into among-species, among-population within species, and among-individual within population components using Arlequin [61]. Analyses were performed considering the whole dataset, and datasets reduced to pairs of species.

##### Within-species analyses to explore the spatial structuring of genetic diversity

Within all populations, genetic diversity was estimated as allelic richness (*AR*; mean number of alleles per locus based on the minimal sample size; [62]) and expected heterozygosity (*H*_E_) using FSTAT. The correlation between population diversity (*AR*, *H*_E_) and latitude was tested through non-parametric Spearman rank correlations in Minitab 12.2 (Minitab Inc., State College, PA, USA). To detect signs of recent bottlenecks, we examined deviations in heterozygosity from mutation–drift equilibrium in each population with the software Bottleneck [63]. We assumed that microsatellite loci follow a two-phase mutation model with 70% single-step mutations and 30% multiple-step mutations.

Within each species, genetic structure was quantified by within-population *F*_IS_ and among-population *F*_ST_ indices using FSTAT, and AMOVAs were used to partition the genetic variance into among-population and among-individual within population components. The statistical significance of *F*_IS_ was assessed by permutations of alleles in each population at each locus, followed by a Bonferroni correction for multiple tests. *F*_ST_ indices were calculated both at the species level and among all pairs of populations. The overall divergence of each population was quantified as the mean pairwise *F*_ST_ between each population and all conspecifics. Exact tests of population differentiation were also performed among all pairs of populations using Genepop [64] and applying a Bonferroni correction (Kopeto was excluded from these tests because of low sample size). We tested the pattern of isolation by distance by performing Mantel tests with 10000 random permutations to compare the genetic and geographic distance matrices. The matrix of geographic distances was calculated from the spatial coordinates of each population using the Geographic Distance Matrix Generator [65]. In order to examine into more details the geographic structure of the genetic diversity, we ran Structure within each species. The analysis parameters were the same as for the multispecies analysis.

Among pairs of species, differences in population *AR*, *H*_E_, *F*_IS_ and overall within-species *F*_ST_ were assessed using permutation tests in FSTAT.

#### cpDNA sequencing

##### Multispecies analyses

We constructed both a haplotype and a population network, using the Neighbor-Net algorithm of Splitstree4 and based on the uncorrected p-distances (i.e. the number of changes between two haplotypes) and pairwise Nst indices (see below), respectively. Using Arlequin, we computed an AMOVA to estimate the proportion of the overall genetic variance found among species, among populations within species, and among individuals within population.

##### Within-species analyses

Within all populations, genetic diversity was estimated as haplotype richness (*HR*; calculated in FSTAT by considering distinct haplotypes as distinct alleles of a single, always homozygous locus) and mean number of pairwise differences between pairs of individuals (*MNbPwDiff*; calculated in Arlequin).

Within each species, among-population differentiation was quantified by *N*_ST_ indices both at the species level and among all pairs of populations using SPAGeDi 1.4 [66]. Compared to *G*_ST_, which only considers alleles/haplotypes frequencies, *N*_ST_ also takes into account the divergence between haplotypes. Here, we used mean number of pairwise differences as a measure of haplotype divergence (similar to uncorrected p-distances and computed in Arlequin), and we performed 10000 permutations of rows and columns of the distance matrix between haplotypes to test whether *N*_ST_ > *G*_ST_. Such a significant relationship suggests that distinct haplotypes are more related within populations than among them, and comparing *N*_ST_ and *G*_ST_ allows assessing the importance of mutation relative to other causes of genetic differentiation (gene flow and divergence time; [67]).

Among pairs of species, differences in population *HR* was assessed using permutation tests in FSTAT, while t-tests were performed in Minitab 12.2 to check for differences in i) *MNbPwDiff* within populations, and ii) *N*_ST_ indices among all pairs of populations within species.

## Results

The five nuclear microsatellite loci displayed 23 to 35 alleles each, leading to 145 alleles observed in total. The overall reproducibility of genotyping was 92%. The concatenated, aligned cpDNA sequences were 1920 base pairs long (respectively 334, 850 and 736 bp for partial *psb*A-*trn*H, partial *trn*S-*trn*fM and *atp*H-*atp*I) and comprised 25 variable sites (4, 16 and 5 respectively) among which 16 nucleotide substitutions and 9 microsatellites displaying 2 to 5 alleles each. This allowed defining 71 haplotypes, each observed in one to 78 individuals.

### Multispecies analyses to examine species divergence and relationships

#### Nuclear microsatellites

**Figure 2:**
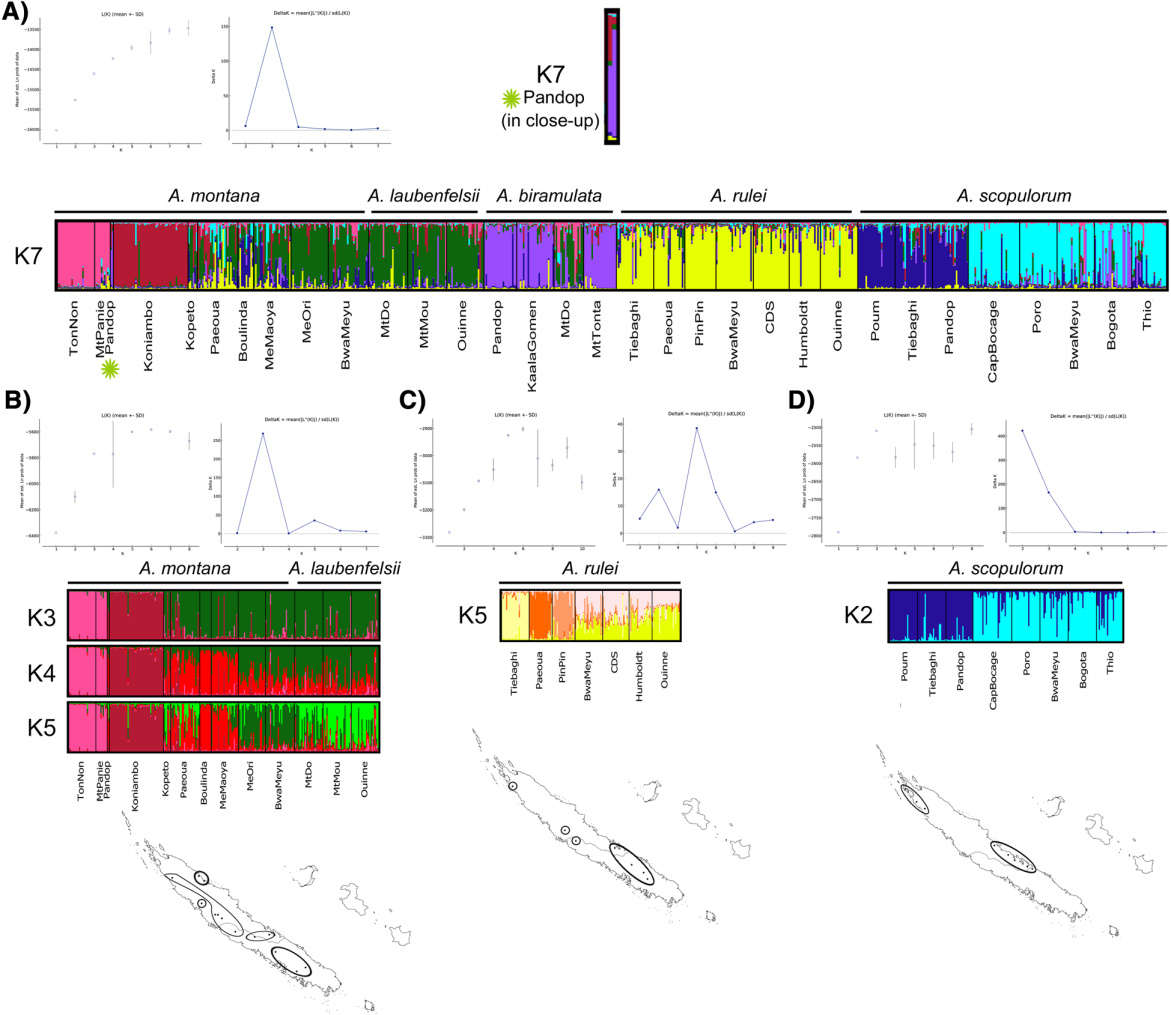
**Results of the structure analyses based on nuclear microsatellites.** For each analysis, the relationships between *K*, L(*K*) and Δ*K* are shown (following Evanno et al. [55] and calculated with StructureHarvester [54]). The cluster partitioning of individuals is presented for the *K*-values that were identified as the most adequate, and discussed in text. Each vertical line represents one individual and the colors represent the membership coefficients to the *K* clusters (displayed with Distruct [68]). Maps show the inferred grouping of populations within species. **A)** On the overall, multispecies dataset. **B)** Within *A. montana*-*A. laubenfelsii*. **C)** Within *A. rulei*. **D)** Within *A. scopulorum*.

In Structure, the probability of the data increased from *K* = 1 to *K* = 8 (Figure 2A). The method of Evanno et al. [55] suggested *K* = 3 as the most adequate number of clusters. However, previous knowledge [34] and other results of this study proved that *A. scopulorum* was the most divergent species included here. Since all *A. scopulorum* populations started to form a distinct cluster, excluding populations of other species, from *K* = 5, we considered the results from this *K*-value. The clustering of populations differed between *K* = 5 and *K* = 6 but, from *K* = 6, remained consistent. In addition, *K* = 7 allowed a clear increase of L(*K*). We therefore considered *K* = 7 as the biologically most plausible number of clusters. The major clusters were: 1) the three *A. scopulorum* populations from the northwestern coast; 2) the five *A. scopulorum* populations from the eastern coast; 3) all *A. rulei* populations; 4) the two northeastern *A. montana* populations (Mt Panie and TonNon); 5) the Koniambo *A. montana* population; 6) the remaining six *A. montana* populations (with the exception of Pandop and with quite strong admixture for the northernmost populations), three *A. laubenfelsii* populations and the *A. biramulata* Mt Do population; 7) the three *A. biramulata* populations Pandop, Kaala Gomen and Mt Tonta and two samples identified as *A. montana* in Pandop (which were admixed with the Koniambo *A. montana* cluster). At the sample level, we observed that some other individuals were admixed, with their genotype originating not only from a cluster characteristic of the species they belong to based on morphological grounds but also, to a large extent, to other clusters: some *A. montana* samples were admixed with the *A. rulei* and/or *A. scopulorum* clusters in Paeoua, Boulinda and Me Maoya; some *A. scopulorum* samples were largely assigned to the *A. biramulata* cluster in Bogota; and a few *A. biramulata* and *A. rulei* samples were largely assigned to an *A. scopulorum* cluster in various sites (Figure 2A).

When the analysis was restricted to *A. montana* and *A. laubenfelsii*, the Mt Panie and TonNon populations on the one hand, and Koniambo population on the other hand were again found to be clearly differentiated from all others (Figure 2B, K = 3). Among the nine remaining populations (excluding Pandop, which was more related to *A. biramulata* based on the previous analysis and not discussed here), two genetic clusters formed a rough genetic cline from north (Kopeto) to south (Ouinee) at K = 4 and, at K = 5, three clusters were retrieved that broadly consisted of the *A. montana* Kopeto-Paeoua-Boulinda-Me Maoya populations, *A. montana* Me Ori-Bwa Meyu populations, and *A. laubenfelsii* Mt Do-Mt Mou-Ouinee populations (Figure 2B).

**Figure 3:**
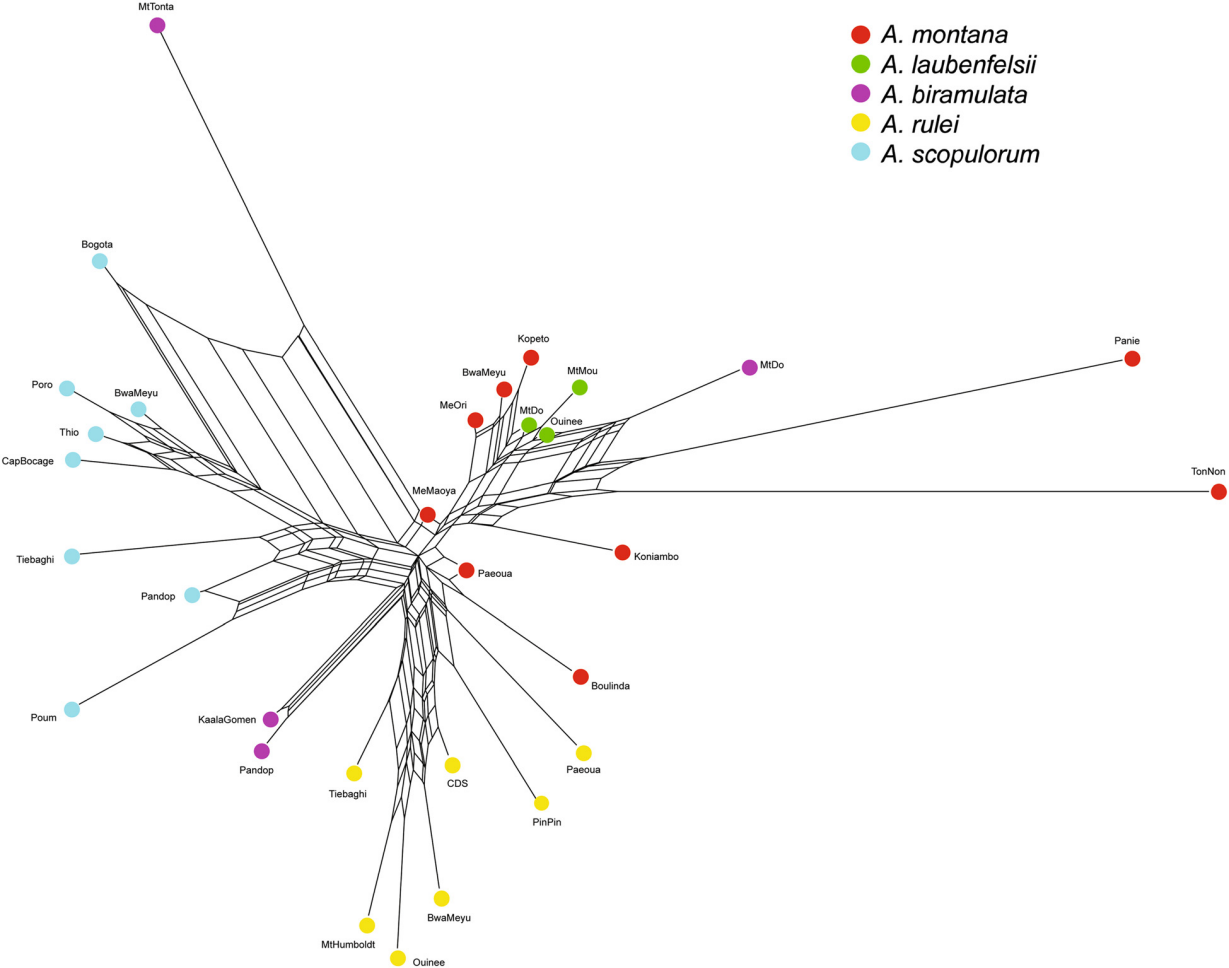
**Neighbor-Net network showing genetic relatedness among the study populations based on nDNA** ***F***_**ST**_ **pairwise indices.** Colours follow the taxonomic identification of populations based on morphology.

The nDNA population network showed the same patterns: short branches suggested strong genetic relatedness among species but *A. scopulorum*, *A. rulei*, *A. biramulata* and *A. montana*-*A. laubenfelsii* formed clearly separate groups (Figure 3). However, there were some exceptions. In particular, the *A. biramulata* Mt Do population was retrieved in the *A. montana-A. laubenfelsii* group (in agreement with Structure) and the *A. biramulata* Mt Tonta population appeared in an intermediate position between *A. montana*-*A. laubenfelsii* and *A. scopulorum*. As in the Structure analysis, we also observed the eastern vs. northwestern differentiation in *A. scopulorum*, and the strong divergence of the Panie, TonNon and to a lesser extent Koniambo populations compared to other *A. montana*-*A. laubenfelsii* populations. The *A. montana* Paeoua and Boulinda populations appeared genetically close to the *A. rulei* Paeoua and PinPin populations, in an intermediate position between the two species.

The overall multispecies nDNA *F*_ST_ was 0.143. The AMOVA showed a low proportion of the genetic variance among species (5.2%), most of it being observed among populations within species (11.5%) and within populations (83.3%). When restricting the analysis to *A. montana* and *A. laubenfelsii*, only 0.4% of the variance was observed between species, which was a much lower proportion than between *A. montana* and other species (Table 3).

**Table 3:** **Results of the AMOVA analyses based on nuclear and chloroplast datasets**

	**nDNA**			**cpDNA**		
	**% var among species**	**% var among populations within species**	**% var within populations**	**% var among species**	**% var among populations within species**	**% var within populations**
*A. montana*/*A. laubenfelsii*/*A. biramulata*/*A. rulei*/*A. scopulorum*	5.2	11.5	83.3	59.8	10.0	30.2
*A. montana*/*A. laubenfelsii*	0.4	10.6	89.0	17.5	12.6	69.9
*A. montana*/*A. biramulata*	1.6	13.4	85.0	55.5	12.6	31.9
*A. montana*/*A. rulei*	4.0	12.0	84.0	49.4	7.7	42.9
*A. montana*/*A. scopulorum*	4.5	12.3	83.2	58.2	10.6	31.2
*A. columnaris*/*A. nemorosa* (calculated from [28])	35.4	4.2	60.5	-	-	-

#### Chloroplast DNA sequencing

**Figure 4:**
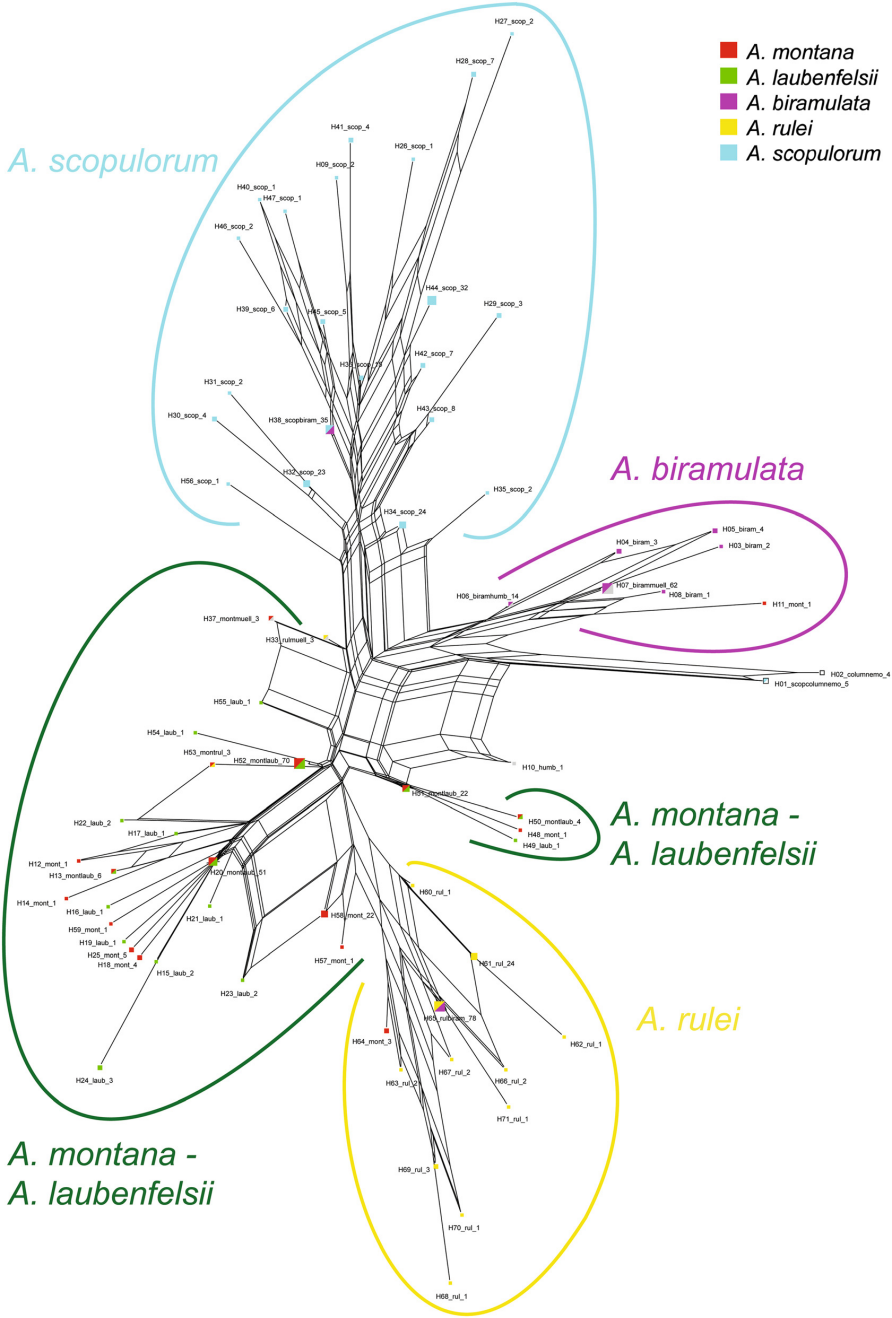
**Neighbor-Net network showing genetic relatedness among the observed cpDNA haplotypes based on uncorrected p-distances.** Each haplotype is designated with its number (H01 to H71), followed by the name(s) of the species and number of samples displaying the haplotype. Colours follow the taxonomic identification of populations based on morphology.

Most cpDNA haplotypes occurred in only one species (Figure 4) and will be referred as ‘specific’ to this species. The remaining 14 haplotypes were shared by several species, and *A. montana* and *A. laubenfelsii* shared as many as five haplotypes while other pairs of species only shared at most one.

In the haplotype network, most haplotypes clustered according to taxonomy (Figure 4). In particular, some samples were retrieved with their conspecifics although the Structure analysis based on nDNA grouped them with another species. It was the case in Mt Do, where all *A. biramulata* samples (except Mt Do_4090) displayed cpDNA haplotypes specific to *A. biramulata* (H05 to H08) although they clustered with *A. montana* and *A. laubenfelsii* based on nDNA (Table 4). In contrast, some individuals that were morphologically and, based on nDNA, assigned to a given species displayed haplotypes that were shared with (or closer to haplotypes observed in) other species (Table 4). All haplotypes of these outlying samples were checked by repeating the PCR reactions. Most importantly, twelve (out of 22) samples of *A. biramulata* from Mt Tonta shared a haplotype that was otherwise only found in *A. rulei* (H65). In contrast, these samples clearly clustered with *A. biramulata* based on nDNA. Also, out of the five samples from Pandop and identified as *A. montana*, we observed that three harboured a cpDNA haplotype specific to *A. biramulata* (H07) whereas two displayed a cpDNA haplotype specific to *A. montana* (H58). All clustered with the *A. biramulata* nDNA cluster. Morphological identification was uncertain and we therefore revised it and considered the three samples with both nDNA and cpDNA related to *A. biramulata* as *A. biramulata* samples in population analyses. The two individuals with cpDNA haplotypes specific to *A. montana* were excluded from population analyses because of the low sample size in this site (n = 2). Other cases of cpDNA *vs.* nDNA incongruence are summarized in Table 4.

**Table 4:** **Incongruent patterns observed between nDNA and cpDNA**

**Population**	**Morphology-based species identification**	**Number of samples studied for cpDNA**	**Number of samples involved in the incongruence**	**cpDNA haplotype specific to:**	nDNA characteristic of*:	**Hypothesized evolutionary scenario (see text)**
Mt Do	***A. biramulata***	19	18	***A. biramulata*** (H05 to H08)	*A. montana-A. laubenfelsii*	Introgression
Mt Tonta	***A. biramulata***	22	12	*A. rulei* (H65)	***A. biramulata***	Introgression (or shared ancestral polymorphism)
Pandop	***A. montana***	5	3	*A. biramulata* (H07)	*A. biramulata*	Erroneous species identification, which was revised to *A. biramulata* for population analyses
			2	***A. montana*** (H58)	*A. biramulata*	Introgression
Paeoua	***A. rulei***	13	1	*A. muelleri* (H33); closer to *A.montana-A. laubenfelsii* haplotypes than to *A. rulei* ones	***A. rulei***	Introgression
Ouinee	***A. rulei***	16	1	*A. muelleri* (H33); closer to *A.montana-A. laubenfelsii* haplotypes than to *A. rulei* ones	***A. rulei***	Introgression
PinPin	***A. rulei***	15	1	*A. montana* (H53); but close (d = 1 change) to the abundant *A. rulei* haplotype H61	***A. rulei***	Population-specific cpDNA haplotype
Panie	***A. montana***	10	3	H64, retrieved with *A. rulei* haplotypes but close (d = 2 changes) to the common *A. montana-A. laubenfelsii* haplotype H52	***A. montana-A. laubenfelsii***	Shared ancestral polymorphism
Paeoua	***A. montana***	16	1	H11, retrieved with *A. biramulata* haplotypes	More related to ***A. montana-A. laubenfelsii*** than to *A. biramulata*	Shared ancestral polymorphism
Mt Do	*A. biramulata*	19	1	*A. scopulorum* (H38)	*A. montana-A. laubenfelsii*	Shared ancestral polymorphism (at cpDNA) and introgression with *A. montana*-*A. laubenfelsii*
Thio	***A. scopulorum***	21	1	*A. columnaris* and *A. nemorosa* (H01)	***A. scopulorum***	Insufficient data to propose an evolutionary scenario

In bold: the species identification upon which two datasets agree (out of the three datasets: morphology, cpDNA and nDNA).

*A sample was considered as characteristic of a given species when > 50% of its genotype was assigned to the genetic cluster(s) of this species.

**Figure 5:**
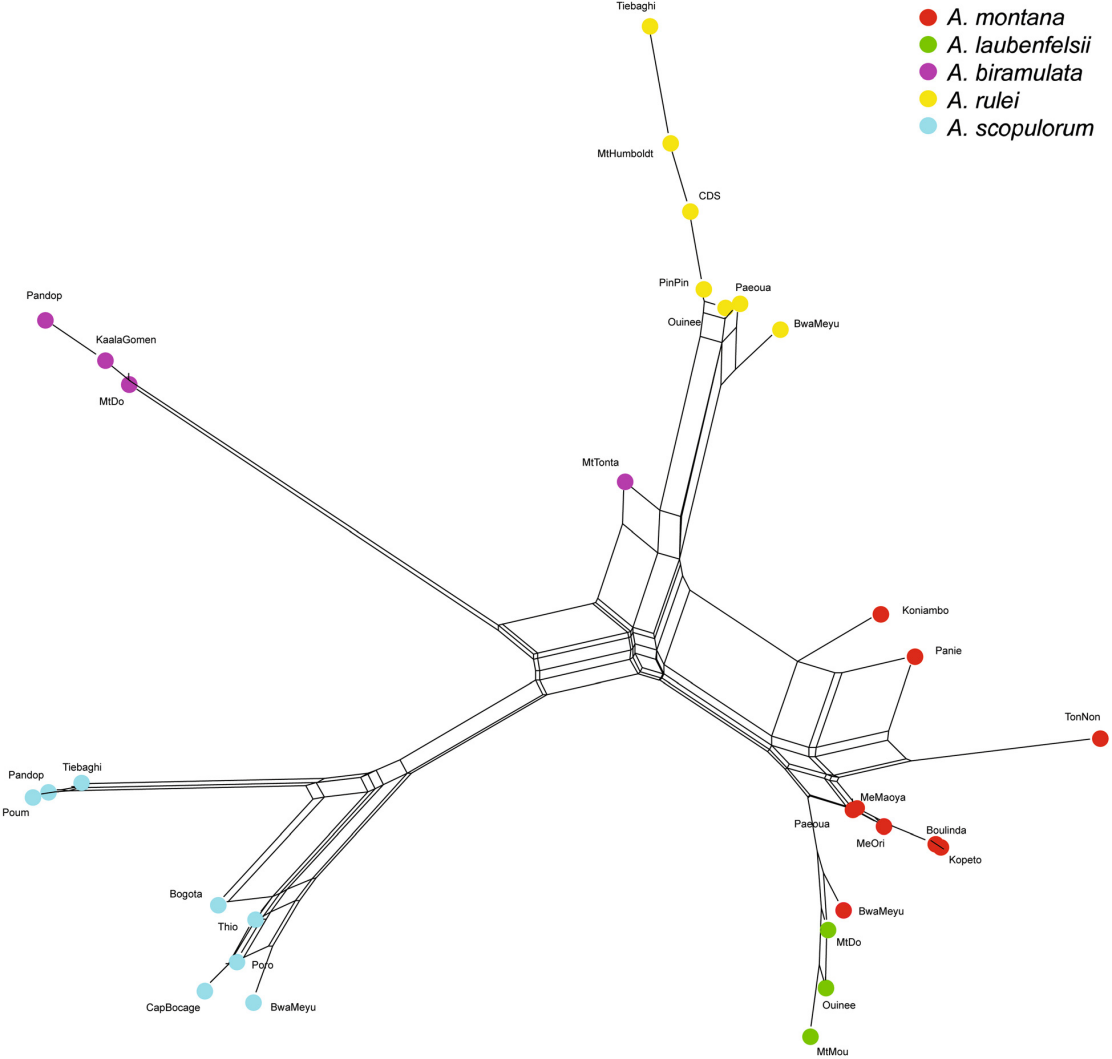
**Neighbor-Net network showing genetic relatedness among the study populations based on cpDNA** ***N***_**ST**_ **pairwise indices.** Colours follow the taxonomic identification of populations based on morphology.

In the population network based on cpDNA data, the distinction among species was clear and even more pronounced than based on nDNA (Figure 5). As with nDNA markers, this was however not true for *A. montana* and *A. laubenfelsii*, whose populations were grouped although the *A. laubenfelsii* populations appeared at the extremity of the *A. montana*-*A. laubenfelsii* portion of the network. Also, the *A. biramulata* Mt Tonta population was retrieved with *A. rulei*, which was expected given haplotype sharing. Similarly to what was found for nDNA, *A. scopulorum* populations separated into two geographic groups.

The overall cpDNA differentiation index *N*_ST_ was 0.646. Consistent with the clear discrimination of species on the population network, the AMOVA showed that 59.8% of the variation was found among species, 10.0% within species among populations, and 30.2% within populations. When considering pairs of species, the among-species component of the variance represented 17.5% of the total variance between *A. montana* and *A. laubenfelsii*, which was much lower than between other pairs of species (most often about 50%; Table 3).

### Within-species analyses to explore the spatial structuring of genetic diversity

#### Differences among species in the levels of genetic diversity and structure

At nuclear loci, there was no significant difference in within-population diversity among species (Table 1), and no population showed signs of a recent bottleneck. For cpDNA, *A. scopulorum* showed the highest levels of population diversity (Table 1). It also displayed larger within-population *F*_IS_ indices than all other species: the mean *F*_IS_ was 0.191 while other species had mean *F*_IS_ < 0.080 when excluding locus Aru1 (*P* = 0.002 among all species; significant between all pairs of species involving *A. scopulorum*; Table 1).

There was no significant difference across species in population differentiation at nuclear loci, with *F*_ST_ ranging from 0.109 and 0.156 (Table 1). AMOVAs also showed similar proportions of the variance among populations across species: 10.1% in *A. montana*-*A. laubenfelsii* and *A. rulei*, 10.4% in *A. scopulorum* and 12.1% in *A. biramulata*. For cpDNA, species-level Nst estimates ranged from 0.108 in *A. rulei* to 0.433 in *A. biramulata* (Table 1). This high value in *A. biramulata* was probably explained by the specific *A. rulei* haplotype exhibited by one part of the Mt Tonta population. *Araucaria montana*-A. *laubenfelsii* and *A. scopulorum* showed intermediate values and, based on Nst estimates among all pairs of populations, displayed significantly stronger differentiation than *A. rulei* (*P* = 0.014 and *P* = 0.003, respectively). The among-population differentiation index Nst was statistically higher than Gst in *A. biramulata* (*G*_ST_ = 0.269, *P* = 0.007; but only based on four populations and with a probably strong bias due to Mt Tonta, see above) and in *A. scopulorum* (*G*_ST_ = 0.174, *P* = 0.001). The relationship was however not significant anymore when we restricted the analysis to the northwestern and eastern parts of the *A. scopulorum* distribution area, respectively. In *A. montana*-*A. laubenfelsii* and *A. rulei*, *G*_ST_ estimates were 0.185 and 0.144.

#### Spatial structure of the genetic differentiation within species

##### In A. montana-A. laubenfelsii *(12 populations)*

CpDNA haplotypic richness was significantly correlated with latitude (*P* = 0.026; *r* = −0.64), with the northernmost populations Mt Panie and TonNon displaying very low diversity and central/southern populations harbouring relatively high numbers of (often private) haplotypes (Table 1). No such geographic trend was observed for other cpDNA or nDNA diversity estimates. At nDNA loci, we detected significant deficits of heterozygotes in Koniambo, Paeoua, Me Maoya and Ouinee (*F*_IS_ = 0.132 to 0.206; Table 1). The Structure analysis based on nDNA suggested the existence of five genetic clusters, which were highly congruent with geography although the Mantel test was not significant (Figure 2B). Two clusters were strongly differentiated and composed of the northern populations Mt Panie-TonNon and Koniambo. Although they belonged to the same cluster, Mt Panie and TonNon were also strongly divergent: they displayed the highest pairwise *F*_ST_ estimate within *A. montana*-*A. laubenfelsii* (*F*_ST_ = 0.315) and split in two distinct clusters at *K* = 6. Other genetic clusters were less clear-cut (and the probability of the data was actually also high for *K* = 3; Figure 2B) but roughly composed of Kopeto-Paeoua-Boulinda-Me Maoya, Me Ori-Bwa Meyu and Mt Do-Mt Mou-Ouinee, respectively. This was congruent with the fact that all populations, except Me Ori-Bwa Meyu and Mt Mou-Ouinee, were significantly differentiated based on exact tests. Based on cpDNA, we also observed the strong divergence of Mt Panie, TonNon and Koniambo, as well as the grouping of the three *A. laubenfelsii* at the extremity of a network branch. The level of population differentiation (as measured by mean pairwise *N*_ST_’s) increased both towards the north and the south of the island, leading to a significant Mantel test (*P* < 0.001).

##### In A. rulei (7 populations)

There was no correlation among diversity indices and latitude. A significant deficit of heterozygotes was detected within Bwa Meyu (*F*_IS_ = 0.131). Based on nDNA, the most adequate number of clusters was *K* = 5, but Structure actually suggested the existence of four genetic groups (Figure 2C). Three of them were composed of only one population each, located in the north: Tiebaghi, Paeoua, and PinPin. The fourth group consisted of the central/southern populations Bwa Meyu, Camp des Sapins, Mt Humboldt and Ouinee. Nevertheless, all populations were significantly differentiated based on exact tests. The cpDNA network also showed the divergence of the northernmost Tiebaghi population (Figure 5). However, there was no correlation between genetic and geographic distances, either for nDNA or for cpDNA.

##### In A. scopulorum (8 populations)

There was no correlation between diversity indices and latitude. Three populations showed significant deficits of heterozygotes: Poum, Cap Bocage and Thio (*F*_IS_ = 0.160 to 0.279; Table 1). Nuclear microsatellites evidenced a clear geographic pattern: in Structure, the most likely number of clusters was *K* = 2 with Poum, Tiebaghi and Pandop forming a northwestern cluster, while Cap Bocage, Poro, Bogota, Bwa Meyu and Thio formed an eastern cluster (Figure 2D). Only two pairs of populations, belonging to the eastern cluster, were not significantly differentiated according to exact tests: Thio-Poro and Thio-Bwa Meyu. The same geographic pattern was clearly observed on the cpDNA network (Figure 5), and the Mantel test was significant in both cases (*P* < 0.001).

## Discussion

In New Caledonia, *Araucaria* species are sometimes difficult to distinguish based on morphology and ecology. Our data showed that genetic markers can allow the revision of taxonomic identifications based on field observations and inspection of herbarium specimens. Moreover, although *Araucaria* trees are emblematic, often dominant in the landscape and supposedly well-known, our results provided new and highly innovative information on species relationships and their evolutionary dynamics.

### Genetics allows distinguishing species –although closely related– except *A. montana* and *A. laubenfelsii*

In contrast to AFLP markers [34], nuclear microsatellites and cpDNA sequencing distinguished most study species. Species differentiation was especially clear based on cpDNA, a pattern that was expected because this genome is haploid, leading to lower effective size and higher differentiation. However, we observed that *A. montana* and *A. laubenfelsii* were not clearly differentiated based on either nuclear or chloroplast data: i) at nuclear loci, they segregated in somewhat different Structure clusters only after some other, conspecific populations were separated; ii) they shared several cpDNA haplotypes; iii) all *A. montana* and *A. laubenfelsii* populations were grouped in nDNA and cpDNA distance-based networks; and iv) the distinction between the two species accounted for only 0.4 and 17.5% of the nuclear and chloroplast total genetic variance, respectively, whereas the distinction between other pairs of species explained three to more than ten times more variance. Using the same five nuclear markers as in the present study, Kettle et al. [28] observed up to 35.4% of the variance between *A. columnaris* and *A. nemorosa*, although the two species belong to the same AFLP clade [34]. *Araucaria montana* and *A. laubenfelsii* therefore seem to belong to a single gene pool. The Structure analysis and cpDNA network suggested that *A. montana* and *A. laubenfelsii* rather form a genetic cline, which may be due to spatial separation given the more southern distribution of *A. laubenfelsii*. The reproductive compatibility between *A. montana* and *A. laubenfelsii* is not known but their morphological distinction appears difficult. Therefore, we suggest that they may be considered as a single species. The subspecies status may nevertheless be justified by their geographic separation and slight genetic differentiation.

### Incongruence between nDNA and cpDNA and nDNA admixture suggest introgression and shared ancestral polymorphism

Interestingly, our results highlighted cases of incongruence between nDNA and cpDNA markers: some individuals possess nDNA predominantly from one species, and a cpDNA haplotype generally associated with another species. Such incongruent patterns could be explained either by gene flow among species (introgression) or by incomplete sorting of ancestral polymorphisms (see e.g. [5,43-45] and references therein).

Reproductive compatibility among New Caledonian species is not known, but our results strongly suggest that hybridization and introgression have occurred in several sites. This is highly probable between *A. biramulata* and *A. laubenfelsii* in Mt Do, and between *A. biramulata* and *A. rulei* in Mt Tonta where individuals combine cpDNA haplotypes of one species with multilocus microsatellite nuclear genotypes of the other species. Such introgression has been repeatedly evidenced between conifer species (see e.g. [69-71]). The most likely scenario is that the species first diversified in allopatry and later came into secondary contact and interbred.

The two species that are suspected of introgression grow in sympatry in Mt Do, which adds credibility to this explanation. The hypothesized introgressing species do not co-occur in Mt Tonta and we cannot exclude the possibility of incomplete sorting of shared ancestral polymorphism (see below). However, *A. rulei* may have been present in Mt Tonta when among-species gene flow occurred, giving rise to the adult trees that we sampled and that are probably > 100 years old. Alternatively, introgression in Mt Tonta may result from occasional long-distance transport of pollen grains since *A. rulei* grows in sites that are only a few kilometres distant (e.g. Mt Humboldt; pers. obs. and [38]). Such introgression between –at least nowadays– allopatric populations has already been reported e.g. between *Picea mariana* and *P. rubens* in North America [71].

Introgression in Mt Do and Mt Tonta also differ in the genetic signature left in the chloroplast and nuclear genomes relative to the morphological identification of the introgressed trees: in Mt Do, trees were morphologically identified as *A. biramulata*, possessed *A. biramulata* cpDNA haplotypes but were predominantly assigned to *A. laubenfelsii* for nDNA. In contrast, in Mt Tonta, trees were morphologically identified as *A. biramulata* (albeit with some morphological traits more typical of –or intermediate with– *A. rulei*) and predominantly assigned to *A. biramulata* for nDNA, but possessed cpDNA otherwise specific to *A. rulei*. Since cpDNA is paternally inherited in conifers, this indicates that *A. biramulata* pollen grains fertilized *A. laubenfelsii* ovules in Mt Do, whereas *A. rulei* pollen grains fertilized *A. biramulata* ovules in Mt Tonta. At nuclear loci, that are biparentally inherited, the genetic composition of the introgressed trees was predominantly influenced by one parental species, as opposed to a mixed influence of the two parents: on average, in Mt Do, the *A. montana-A. laubenfelsii* clusters contributed 76% to the nuclear genotype of the introgressed individuals, whereas the *A. biramulata* cluster contribution was only 15% (although three samples had similar contributions of the *A. biramulata* and *A. montana* clusters, and one sample had a predominant contribution of the *A. biramulata* cluster). In Mt Tonta, the *A. biramulata* cluster was largely predominant in the genotype of all introgressed trees, and contributed as much as 88% to their genotype on average. This suggests, first, that most introgressed trees that we sampled do not belong to the F1 generation, which would show equal contributions from the two parental species, and that hybridization is more ancient. Second, it implies that the initial hybrids backcrossed much more extensively with one parent than with the other one: hybrids would have mostly backcrossed with *A. laubenfelsii* in Mt Do, and with *A. biramulata* in Mt Tonta. This is congruent with the relative abundance of the parental species in the two sites, since *A. laubenfelsii* is dominant in Mt Do (based on our field observations) and *A. rulei* was not recently observed in Mt Tonta. At each generation, the proportion of the other parent genome in the nucleus of the introgressed tree was halved, ultimately leading to a very low contribution. It is intriguing that such extensive backcrossing with *A. laubenfelsii*, in Mt Do, gave nevertheless rise to trees that were morphologically identified as *A. biramulata.* However, only a very small number of loci may be crucial in bringing about the morphological differences that distinguish the species, and the five loci used here may not be involved in such differences [43].

Hybridization and introgression may also have happened between *A. montana*-A*. laubenfelsii* and *A. biramulata* in Pandop (as suggested by the nDNA vs. morphology and cpDNA incongruence of two *A. montana* samples) and between *A. rulei* and *A. montana*-*A. laubenfelsii* in Paeoua and Ouinee (as suggested by the cpDNA vs. morphology and nDNA incongruence in one *A. rulei* sample in each site). The involved species co-occur in these geographic locations and, in Pandop, the larger assignment of the putative introgressed samples to *A. biramulata* compared to *A. montana*-*A. laubenfelsii* based on nuclear data is in agreement with the higher abundance of *A. biramulata* in this site. A lower degree of confidence is associated with the possible cases of introgression in Paeoua and Ouinee, because the putative introgressed samples do not display full genetic (cpDNA haplotype) identity with their potential paternal species *A. montana*-*A. laubenfelsii*, but only close genetic relatedness. However, it is interesting to note that the population network based on nDNA data indicated close genetic relatedness between the *A. montana-A. laubenfelsii* and *A. rulei* populations in Paeoua, and that the Structure analysis showed nuclear admixture between the two species at this site. This would be congruent with the occurrence of hybridization and introgression. Introgression may also have happened in Boulinda, where both species also co-occur and where A*. montana*-*A. laubenfelsii* samples appeared genetically close to *A. rulei* based on the nDNA population network, and admixed with *A. rulei* at nuclear loci based on the Structure analysis.

Introgression may also explain cases of nuclear admixture observed at other geographic locations, although the involved species do not always co-occur. This would suggest a high potential for pollen dispersal across sites, and deserves further investigations.

Another possible explanation for incongruence between chloroplast and nuclear data is the incomplete sorting of shared ancestral polymorphism in one of the two genomes. It is more likely at loci exhibiting a lower degree of variation, and therefore much more plausible in DNA sequences of cpDNA intergenic regions than at nDNA microsatellite loci. Shared cpDNA haplotypes were observed between *A. montana*, *A. biramulata*, *A. rulei*, *A. muelleri* and *A. humboldtensis*, between *A. columnaris* and *A. nemorosa*, and even between species that do not belong to the same AFLP clade (*A. scopulorum*-*A. biramulata*, and *A. scopulorum*-*A. columnaris*-*A. nemorosa*). Some other haplotypes that were previously shared may have gone extinct in some species while not in others. This may be the case e.g. for the *A. montana* haplotypes H11 in Paeoua and H64 in Mt Panie. Shared ancestral polymorphism suggests that species diverged relatively recently, have large effective sizes (delaying the fixation of polymorphisms) and/or experience slow rate of cpDNA sequence evolution [1,46].

### Comparative within-species phylogeography: a shared pattern of pronounced population differentiation, in tight relation to their spatial distribution

We observed a slightly stronger within-species genetic structure than commonly found in outcrossing, wind-pollinated conifers: nuclear *F*_ST_ indices ranged from 0.109 to 0.156 and 10.1 to 12.1% of the total nDNA variance was observed among populations, depending on the species, whereas Hamrick et al. [72] reported an average *F*_ST_ of 0.073 for various gymnosperms based on allozymes and Petit et al. [73] reported an average *F*_ST_ of 0.116 at various biparental markers in conifers. This is most likely explained by the fact that New Caledonian *Araucaria* species do not conform to the general model of conifers growing as large, wide-ranging populations with no physical barriers over long distances, which usually leads to high gene flow and low differentiation in temperate, northern Hemisphere species (e.g. [74]). In agreement with our results, similar high levels of population differentiation were found in some other tropical conifers (e.g. [75] and references therein) and notably in other *Araucaria* species: based on RAPD markers, Bekessy et al. [76] estimated that 12.8% of the total genetic variance was found among populations of *A. araucana*, Pye et al. [77] reported this proportion to be 20.3% in *A. cunninghamii* and Pye and Gadek [78] observed as much as 37% of the variation among populations of *A. bidwillii*. In *A. angustifolia*, 19.0% of the AFLP variance was retrieved among populations [79]. The only estimate based on microsatellite markers in the genus was an among-population variation component of 11.2% in *A. angustifolia* [80]. At chloroplast loci, Schlögl et al. [81] estimated *G*_ST_ = 0.280 in *A. angustifolia*, which is slightly higher than our estimates spanning from 0.144 to 0.269. Differentiation levels observed in *A. montana-A. laubenfelsii*, *A. biramulata*, *A. rulei* and *A. scopulorum* were higher than those observed in *A. columnaris* and *A. nemorosa* using the same microsatellite markers (*F*_ST_ of 0.052 and 0.060 in *A. columnaris* and *A. nemorosa*, respectively; [28]). This must at least partly be due to the smaller among-population spatial distances in the latter two species. Here, the relatively strong population differentiation could be related to: i) the complex topology of New Caledonia –with most populations growing in mountain areas separated by valleys– and low pollen and seed dispersal abilities, resulting in low gene flow and long-term isolation of populations; and ii) the occurrence of inbreeding, evidenced by significant *F*_IS_ indices, which strengthens the influence of genetic drift.

For all three species examined, we observed a clear geographic structure of the genetic diversity. Three main scenarios could explain this. First, it may result from contemporary gene flow occurring primarily between neighbouring populations. However, rather high *F*_ST_ values, significant differentiation of spatially close populations (e.g. the *A. montana* populations of Paeoua and Kopeto and *A. scopulorum* populations of Thio and Bogota, separated by a distance of less than 5 and 10 km, respectively) and the non-significance of most Mantel tests offer limited support to this hypothesis. Second, different genetic clusters could correspond to groups of populations originating from distinct refugia, where the species would have survived periods of adverse environmental conditions and from which they would have colonized their current geographic ranges when conditions became suitable. A retreat of (at least some) *Araucaria* species towards refuge areas probably happened during the Quaternary climatic oscillations and genetic divergence could therefore result from prolonged isolation. More genetic, paleontologic and paleoclimatic data are needed to propose hypotheses on the timing and location of potential refugia [30-32]. Third, current populations may be remnants of an ancient, more or less continuous population and intermediate, now extinct populations could have acted as stepping stones for gene flow. This would explain the non-significant difference between *N*_ST_ and *G*_ST_ in *A. montana*-*A. laubenfelsii* and *A. rulei* (and *A. scopulorum* when considering northwestern and eastern populations separately, see below). Subsequent fragmentation of the ancestral population could have occurred in response to e.g. competition, erosion of ultramafic soils (that once covered the entire island but now cover only a third of the surface [11]), or more recent human-induced habitat destruction. On the overall distribution area of *A. scopulorum*, we found *N*_ST_ > *G*_ST_. This suggests that gene flow and common ancestry are not sufficient to counteract the divergence of populations due to mutation. Such a pattern is not unexpected given the highly disjunct distribution of the species, which makes gene flow very unlikely between northwestern and eastern populations.

Some populations displayed especially strong divergence, and the case of the *A. montana* populations of Mt Panie and TonNon was particularly striking. The divergence of the Mt Panie population was previously reported [34]. The TonNon population grows in the same mountain massif, and the two sites were also characterized by low levels of genetic diversity. A founder effect following long-distance dispersal appears unlikely, because populations would be expected to display close relationships with their population(s) of origin, which was not observed. The two populations may originate from specific refugia (possibly two distinct refugia since they are also clearly differentiated from each other), which did not act as sources for colonization elsewhere. They are also spatially very isolated, which likely results in low potential for gene flow and can account for their divergence and low diversity because of genetic drift. In addition, these populations differ by the acidic substrate onto which they grow since all others are found on ultramafic substrates. This could have limited gene flow to even lower levels if the different type of soil prevents the establishment of non-adapted seeds that would migrate from other (ultramafic) sites. We therefore suggest that differential selection in response to distinct soil substrates may have contributed to an even stronger genetic divergence at neutral markers.

At the population level, we observed significant deficit of heterozygotes in eight populations out of 31. This could be due to the occurrence of null alleles, but we did not observe PCR failure at just one particular locus, as expected in individuals that are homozygous for a null allele. Therefore, the deficit of heterozygotes may rather be explained by the existence of geographic substructure within populations (Wahlund effect) due to selfing or biparental inbreeding following reproduction among related individuals. Such biparental inbreeding was previously shown in *A. nemorosa* [28] and the dioecious *A. araucana* and *A. angustifolia* [80,82].

### Conservation implications

In the absence of additional data, we suggest that *A. montana* and *A. laubenfelsii* may be considered as a single evolutionary unit in conservation plans. In contrast, we observed a clear differentiation of the northernmost *A. montana* populations of Mt Panie and TonNon. This may be related to different soil adaptations, and suggests the need to protect these populations as they could represent a distinct Evolutionary Significant Unit (ESU; [83]).

Our data showed strong signs of introgression, involving *A. biramulata* in particular. Such ancient hybridization events and introgression can be seen as a motor of evolution, potentially given rise to evolutionary novelties [84]. However, *A. biramulata* grows in only a few sites and the risk of genetic assimilation by more widespread species should be carefully monitored and minimized in order to maintain the species [85].

Last, the negative effects of recent population fragmentation were not detected in the genetic structure of contemporary adult populations, but they may be observed in the future [28,86]. Therefore, it appears crucial to protect the habitat of these species that are often found on (or in the immediate vicinity of) open-cast mining sites.

## Conclusions

New Caledonian *Araucaria* species form a complex of closely related species, which probably diverged recently and/or are characterized by a slow rate of molecular evolution, and among which ancient hybridization and introgression is strongly suspected. Future studies on the evolutionary dynamics of this group should adopt an integrative approach –combining e.g. genetics, ecology and morphology– and simultaneously consider multiple species given their tight relationships and probable gene flow [87]. Mitochondrial markers would be highly valuable since mtDNA is only dispersed by seed movement –which is likely more restricted than pollen movement– and is therefore expected to retain earlier geographic structure [73,88]. In order to examine the impact of environmental features on the distribution of genetic diversity within New Caledonia, as many species as possible should be surveyed in a population genetic/phylogeographic framework to allow comparisons and the identification of general trends (comparative phylogeography). The divergence between *A. montana* and *A. laubenfelsii* should also be assessed based on morphological and ecological characters, in order to confirm or reject our hypothesis that they form a single species. Within *A. montana*, the potential adaptation to different soil substrates (acidic vs. ultramafic) and its influence on genetic differentiation would deserve attention. Last, the hybridization and introgression processes offer many exciting questions to tackle, e.g. how are the introgressed individuals related to their parental species in terms of morphology and ecology? When did hybridization occur, how common were such events and do they still occur nowadays? Could they lead to the emergence of new, self-sustainable and reproductively isolated hybrid species?

## Additional file

Additional file 1:
**Geographic location and vouchers of the study populations.** Herbarium vouchers were deposited at the Herbarium of the Royal Botanic Garden Edinburgh, UK (E).
